# Supplementary material for: Comparative Mitogenome Analyses Uncover Mitogenome Features and Phylogenetic Implications of the Reef Fish Family Holocentridae (Holocentriformes)
Source: Biology (Basel). 2023 Sep 22;12(10):1273. doi: 10.3390/biology12101273 (PMC10604132; doi:10.3390/biology12101273)

Myripristis murdjan

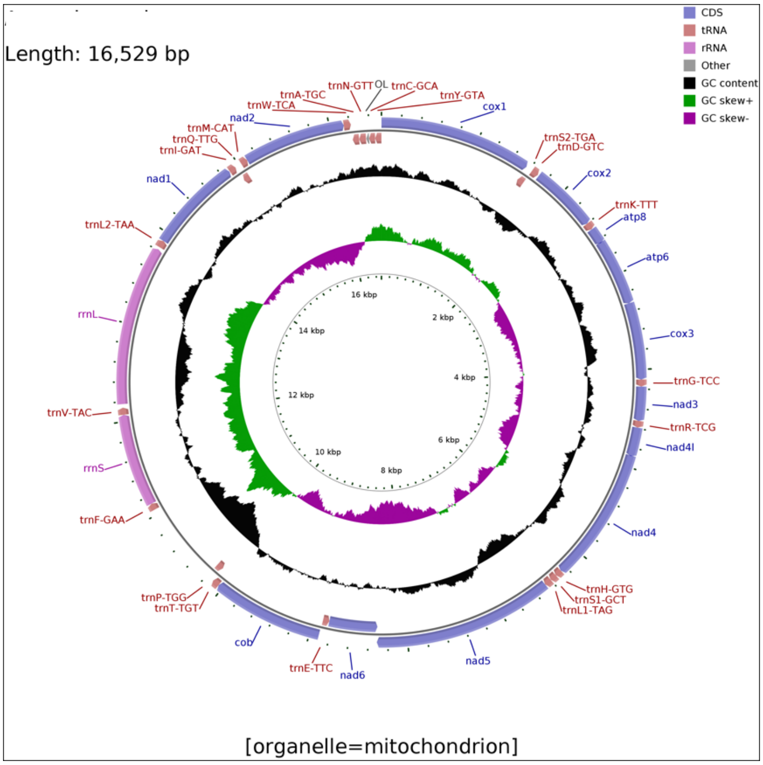

Myripristis violacea

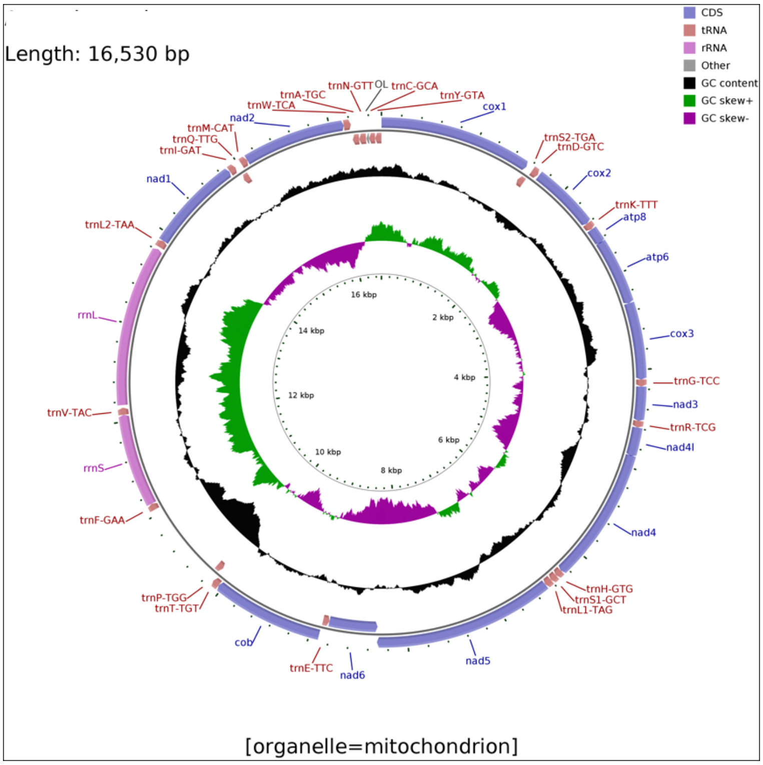

Neoniphon opercularis

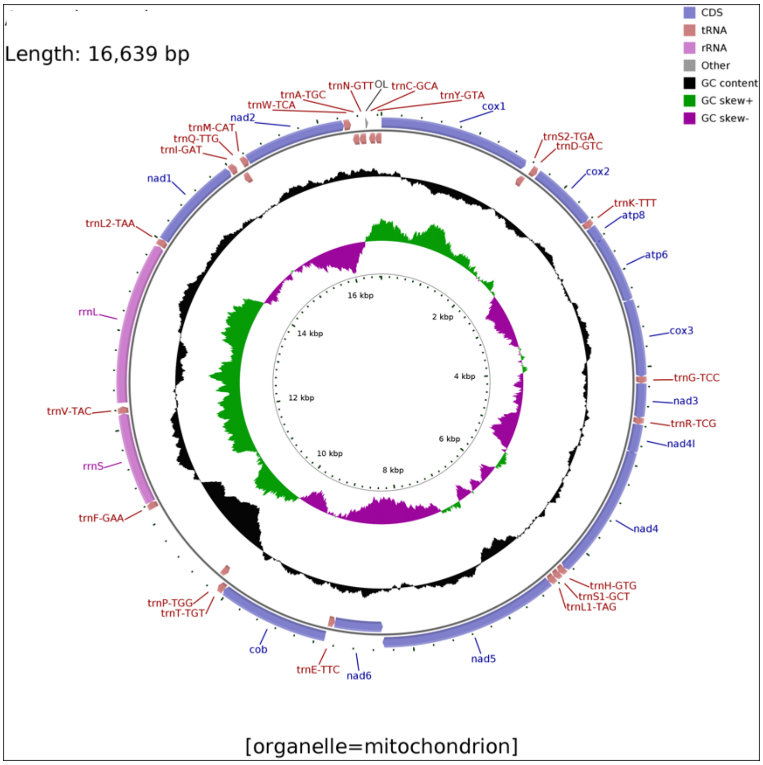

Sargocentron caudimaculatum

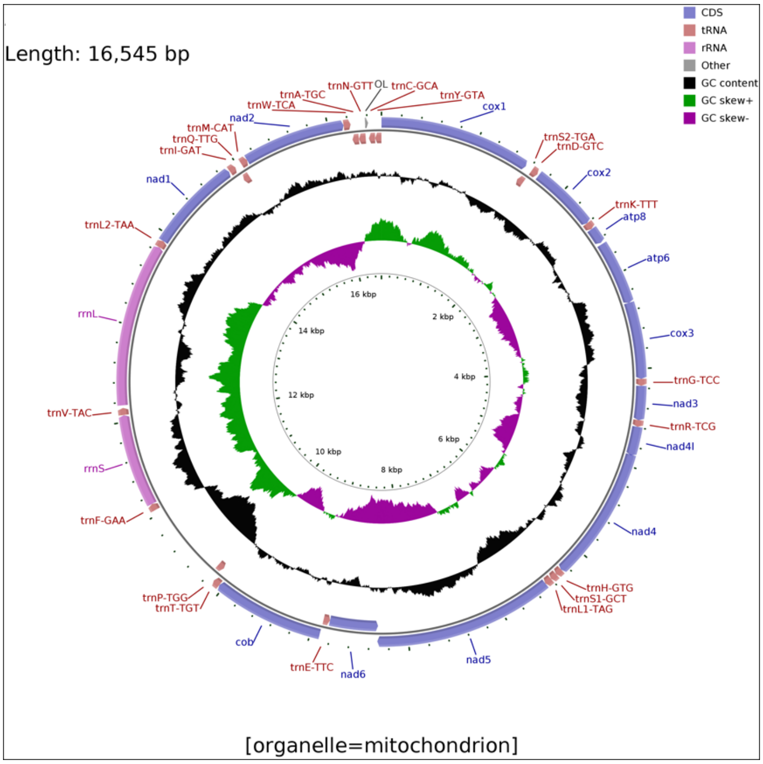

Sargocentron diadema

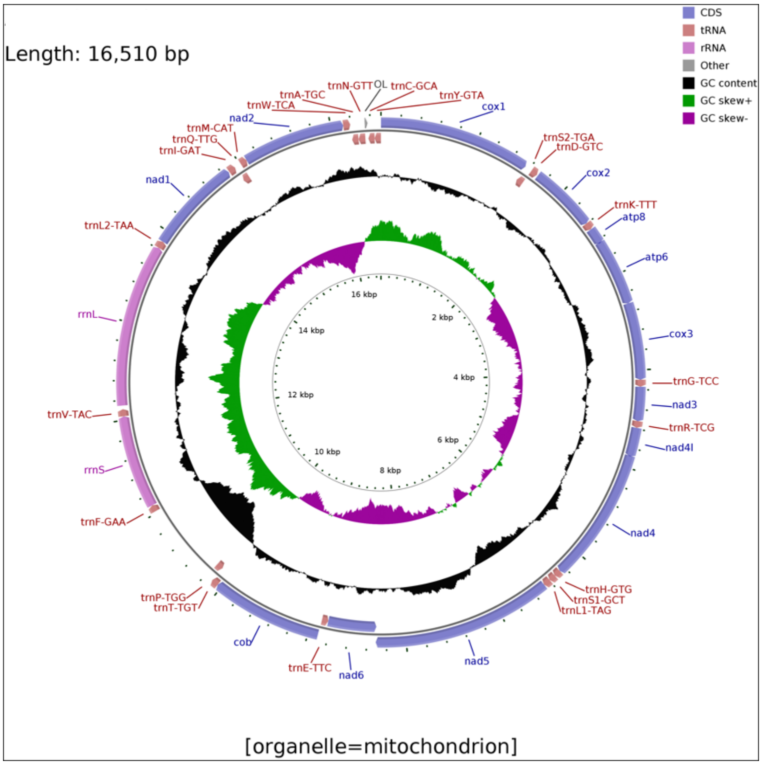

Sargocentron melanospilos

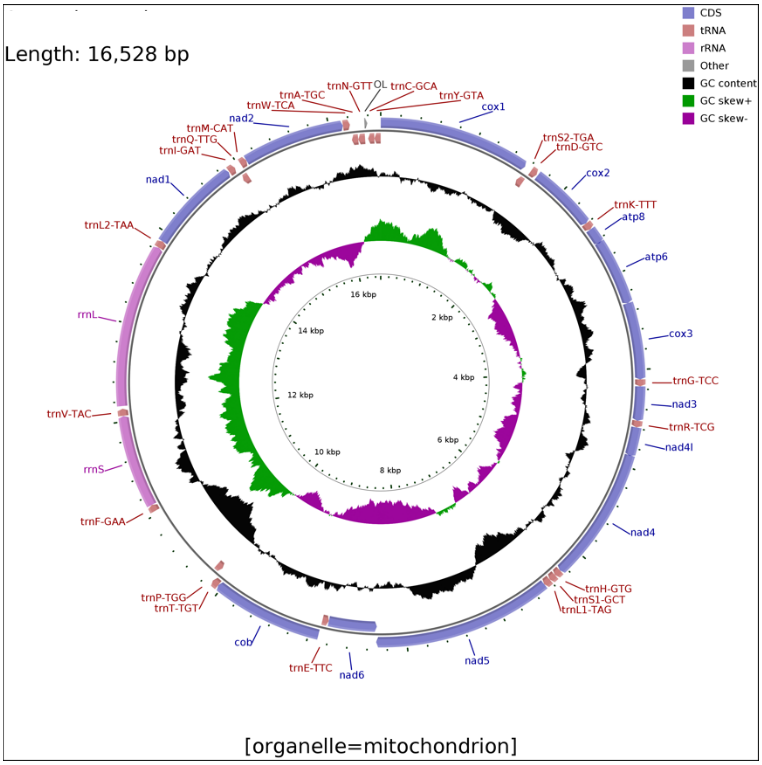

Sargocentron punctatissimum

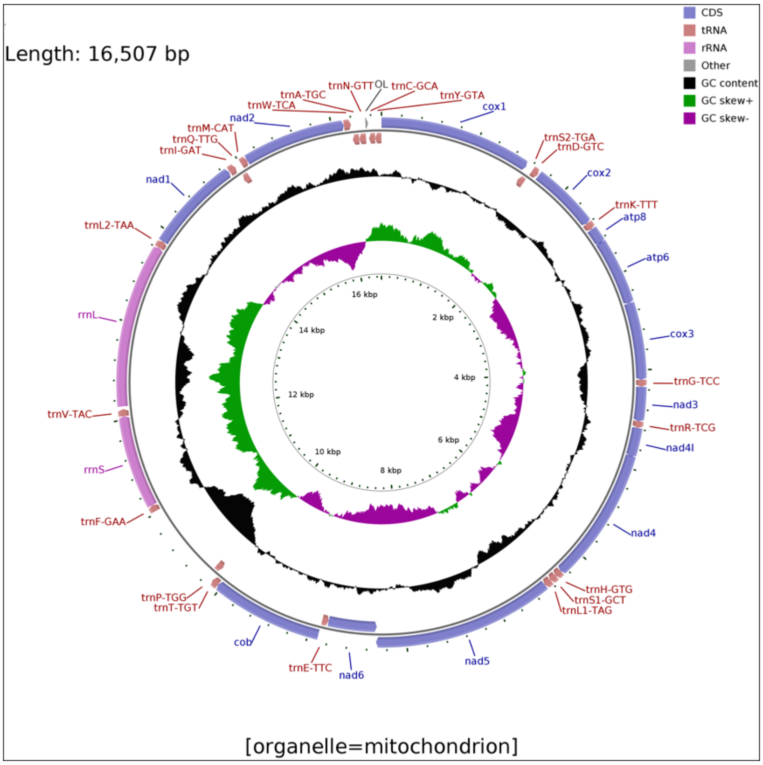

Supplement: Supplementary file 1 [file biology-12-01273-s001.zip › Figure S1.pdf]
