# Supplementary material for: Comparative Mitogenome Analyses Uncover Mitogenome Features and Phylogenetic Implications of the Reef Fish Family Holocentridae (Holocentriformes)
Source: Biology (Basel). 2023 Sep 22;12(10):1273. doi: 10.3390/biology12101273 (PMC10604132; doi:10.3390/biology12101273)
Supplement: Supplementary file 1 [file biology-12-01273-s001.zip › Figure S2.pdf]

```
Sargocentron_caudimaculatum      --------------------------------------------------
Sargocentron_spiniferum          --------------------------------------------------
Sargocentron_melanospilos        ------------------------------------------AACCTCCA
Sargocentron_rubrum              --------------------------------------------ACTCCA
Neoniphon_sammara                -----------------------------------------TTATATAAT
Sargocentron_diadema             --------------------------------------------------
Sargocentron_punctatissimum      --------------------------------------------------
Neoniphon_opercularis            ---------------------------------ATCTACAACAAACTATC
Myripristis_kuntee               -------------------------------------ACTTTTCAGACAT
Myripristis_violacea             -------------------------------------ATTTTTCAGACAT
Myripristis_murdjan              -------------------------------------GTTCAACAGACAT
Myripristis_berndti              --------------------------------------TCCAACAGACAT
Myripristis_vittata              ----------------------------------------TTTTATACAT
Ostichthys_japonicus             CTTCCAGAATAAATGCCCACACATCTATTTATTAATGCGGTTGTAGACAT

Sargocentron_caudimaculatum      ------AACTACG--CCACGCCAAA-TTAAGAGAAAACATACGAGTAACA
Sargocentron_spiniferum          -------ACTACG--CCACACCAAA-TTAAGAGAAAACATACGAGTGATA
Sargocentron_melanospilos        AACAACCACACCG--ACACATAAAA-ATAAGGGCAAACACTCGAACAACG
Sargocentron_rubrum              AACAACCACACCG--ACACATAAAA-ATAAGGGCAAACACTCGAACAACG
Neoniphon_sammara                ATTATTTACCCCAATCTATAGCTAC-AAAAGAGAAAACACCCAAGCGACA
Sargocentron_diadema             -------ACTTTAA-AAACCTCAAA-CTTAAGGAAAAGCCTCGAGCGACA
Sargocentron_punctatissimum      ---ATTTCCACGACCTCTTATCAAACTTAAAGGGAATTTCCCCGACAATA
Neoniphon_opercularis            TGTAATTCTTTACACAATTTTATAGTTTGAACGGGAAAGTCCAAGTAGCG
Myripristis_kuntee               ATATACAATATTACCACATACTG-TACTTAAAACAATACACCCAATGTTC
Myripristis_violacea             ATATACAATATTACCATATACTG-TACTTAAAACAATACACCCAATGTTC
Myripristis_murdjan              ATATATAATATTACCATATATTG-TACT-AAAACATTACATGTAATGCTC
Myripristis_berndti              ATATACAATATTACCATATATTG-TACT-AAAACATTACATGTAATGCTC
Myripristis_vittata              ATATACAATATCACCATATAATGATATCAAGTGCATTAAATGTAATGCTT
Ostichthys_japonicus             ATATGTATTATTACCATACATTTATATTAAACATATAATATGTAATGCTT
                                                              *

Sargocentron_caudimaculatum      CGCA--ATGCTATGTATT--AACACCATTCGT-CGGTACACACCATTCAG
Sargocentron_spiniferum          CGCA--ATGCTATGTATT--AACACCATTCGT-CGGTACACACCATTCAG
Sargocentron_melanospilos        CCAAC-GCGCTATGTATT--TACACCATTTAT-TGGTGTTAACCATACAG
Sargocentron_rubrum              CCAAC-GCGCTATGTATT--TACACCATTTAT-TGGTGTTAACCATACAA
Neoniphon_sammara                ATAAC-TAACTATGTATT--TACACCATATAT-TGCTATACAATCCACAG
Sargocentron_diadema             AC-AT-CCGCTATGTATTA-TACACCATATAT-TTATATTAACCATTCAG
Sargocentron_punctatissimum      AGATTTGAGCTATGTATT--ATCACCATACAA-TTATATTAACCATACAG
Neoniphon_opercularis            AATAC-AAACTATGTATT--TACACCATATAT-TGATATTAACCCCACAG
Myripristis_kuntee               TAAATACA-GTATATGTATTTACACCATATCA-TTATATTAACCATTCAA
Myripristis_violacea             TAAATACA-GTATATGTATTTACACCATACCA-TTATATTAACCATTCAA
Myripristis_murdjan              TCAAAACATACATATGTAATAACACCATAACGCTTATATTAACCACTCAG
Myripristis_berndti              TTAAGACATACATATGTAATAACACCATAACATTTATATTAACCATTCAA
Myripristis_vittata              ATAAACATAATATATGTTATTTCACCATTAAA-TTATTTTAACCATTCAA
Ostichthys_japonicus             AAGGACA-AATATATGTATTATAACCATTGAT-TTATATTAACCATTCAA
                                            ** * *      *****        *    *     **
```

```
Sargocentron_caudimaculatum      GTAATACTA-----TCG-ACTAATATA--AATTTTACATAAACCATTA--
Sargocentron_spiniferum          GTAATACTA-----TCG-ACTAAATCA--AACTTTACATAAACCATTA--
Sargocentron_melanospilos        GTAATATTACAATATTC-ATGATTCCA--CATAGTACATTTTAAATTAC-
Sargocentron_rubrum              GTAATATTACAATATTC-ATGATTCCA--CATGGTACATTTTAAATTAC-
Neoniphon_sammara                GTAATACTAGTTAACTA-AATATATTT--CATAATACATTTATATTTACC
Sargocentron_diadema             GTAATACTAAATTATGATATAATTGTA--CATAAACCAGTTATTGTAA--
Sargocentron_punctatissimum      GTAATACTAG-----TAAAACCTTAAT--TATTTTACACACAAATACAT-
Neoniphon_opercularis            GTAATACTAATAAATATGCTTAATATTTGCATAATACATATTAAATAATA
Myripristis_kuntee               GAAGTACA-TTAAAATG-CTTAATTT-TACATAATACATTC-AAATCTT-
Myripristis_violacea             GAAATACA-TTAAAATG-CTTAATTT-TACATAATACATTC-AAATCTT-
Myripristis_murdjan              GGAACAGA-TAGAAACG-CTTAATTT-CCCATAAGACATTTCAAGTTCC-
Myripristis_berndti              GGAATGGAGTTAAAACG-CTTGATCTATCCATAAGACATTT-AAACTTT-
Myripristis_vittata              GGAATAAA-TGCGACCT-CATAATCT-TACATTATACATTA-ATACTCT-
Ostichthys_japonicus             GAAATAAC-AGTCAAGT-AA-AACAT-TACATAATACA--A-TTACATA-
                                 * *                           *     **

Sargocentron_caudimaculatum      AA-ATATAAAATATTGA------ATTCTAAAGTAACGGGA---CCATA-T
Sargocentron_spiniferum          AA-ATATAAAATATTGA------ATTCTAAAGTAACGAGA---CCATT-T
Sargocentron_melanospilos        AA-GTATGAAGACATAA------AAATGATATCAAAGGAA---ATATGGT
Sargocentron_rubrum              AA-GTATGAAGACATAA------GAATGGTATCAAAGGAA---ATATGGT
Neoniphon_sammara                GA-GAACGGGAACATAAC-----AAGTTAAATCAAGGAAATTTCCCTTAA
Sargocentron_diadema             AA-ATCCATAAATATCAT-----GAATTCAAGTGACGGGA---CCATA--
Sargocentron_punctatissimum      TG-GTTTAAATAGATCAC-----CATGCGAATAGTATGAA--AGTATGTT
Neoniphon_opercularis            AATATACATAATAATTAATTACAGACTAACATTAAAAGATTATTTATGAG
Myripristis_kuntee               ---TATTTCATATATAAT----------CAATTCAAGGAA----TATAAT
Myripristis_violacea             ---CATTTCACATATAAT----------TAATTCAAGGAA----TATAGC
Myripristis_murdjan              ---AGCCTCAGGCA-GATA--------CTGATTTAAGAGA----TA-AGT
Myripristis_berndti              ---AGCTCCAGGCATGACA--------CTGATTTAAGAGA----TA-AGT
Myripristis_vittata              ---TTATTCACCCCAGAT------------ACTCATCATC----CACTGT
Ostichthys_japonicus             ---TTCATAACCATTCGT------------AGTAA--AAT----AACTGT
                                                               *

Sargocentron_caudimaculatum      ACTTGA-------------AATATACA-------------ACTAAAAAAA
Sargocentron_spiniferum          ACTTGA-------------AATAAACA-------------ACTAAAAATA
Sargocentron_melanospilos        TCGTAA-------------AATCAAGA-------------CCTAGCATAA
Sargocentron_rubrum              TCGTAA-------------AATCAAGA-------------TCTAGCATAA
Neoniphon_sammara                AAGTAA-------------GTTTAAGA-------------CCGAGCTATT
Sargocentron_diadema             ---TAT-------------AATAAAAT-------------TTTAACAGAT
Sargocentron_punctatissimum      TGAAAG-------------ATTTAAGA-------------CCGAACACAT
Neoniphon_opercularis            TCTTAACCAATATACAAGTATTTAATAGACATATATTATTTCTACTGAAT
Myripristis_kuntee               ACTAGT-----------AAACTCCACA-------------CCTAACATAA
Myripristis_violacea             ACTAGT-----------AAACTCCACA-------------CTTAACATAA
Myripristis_murdjan              ACCAGA-----------ACACA--AGA-------------CCTAACAGGA
Myripristis_berndti              ACTATA-----------AAATA--AGA-------------CCTAACAATG
Myripristis_vittata              ATACAC-----------GGATTTAAGA-------------CCTAACACA-
Ostichthys_japonicus             AACCAA-----------AAATTTAAGA-------------CCTAACACTT
                                                         *                  *
```

```
Sargocentron_caudimaculatum      TGAAATCA--AATGGCAG--ATATACCAAGTAAT-CACCATTACAAGTGA
Sargocentron_spiniferum          TGAAATCA--AATAGCAG--ATATACCAAGTAAC-CACCATTACAAGTGA
Sargocentron_melanospilos        AGAAATCAT-GGCCAAAG--ATATACCAAGTCCC-CACCATATTA--TGA
Sargocentron_rubrum              AGAAATCAT-GGCCAAAG--ATATACCAAGTCCC-CACCATACTA--TGA
Neoniphon_sammara                CAAACTTATAAGATAAAG--ATATACCAAGTCCC-CAACATCTCGTCATA
Sargocentron_diadema             T-ATATCATCAGTCA-AG--ATATACCAAGTAAT-CCACATCCTGTTATA
Sargocentron_punctatissimum      TTAATTCATCAGTTT-AG--ATATACCAAGTAAT-CAACATTCTATAATA
Neoniphon_opercularis            TGAACCTACAAGTAACAAGAACGTACCAAGTAACTCAACATCCCGTTATG
Myripristis_kuntee               AAGTTATTTTAAC--CAG--ATATACCAAGTAAT-CACCATTCTATTTCA
Myripristis_violacea             CAATTATTTTAAC--CAG--ATATACCAAGTAAC-CACCATCCTACTTCA
Myripristis_murdjan              AGAAGAAATCGATTGCAC--ATATACCAAGTACT-CAACAATCTATTT-A
Myripristis_berndti              GAAAGAAATCGATTGCAT--ATATACCAAGCACT-CAACAACCTATTT-A
Myripristis_vittata              ATATTCATTAGTC--AAG--ATATACCAAGCACC-CAACATCCGGCC---
Ostichthys_japonicus             AAACCCATTAGTC--AAG--ATATACCACGTACC-CACCATCCCGTT---
                                                 *   *  ***** *     *  **

Sargocentron_caudimaculatum      AAATCACAT--TTTTAATGTAGTAAGAAACCACCAAAG-AATGATTACTA
Sargocentron_spiniferum          AAATCGCACCATATTAATGTAGTAAGAAACCACCAAAG-AATGATTACTA
Sargocentron_melanospilos        GATTAAAGGAATCTTAATGTAGTAAGAGACCACCAAAG-AATGATTTCTA
Sargocentron_rubrum              GATTGAAGGAATCTTAATGTAGTAAGAGACCACCAAAG-AATGATTTCTA
Neoniphon_sammara                AAATT-CAGAACTTTAATGTAGTAAGAAACCACCAAAA-TTTGATTCCTG
Sargocentron_diadema             AAACTTAACTAT-TTAATGTAGTAAGAAACCACCATAA-ATTGATTTCTT
Sargocentron_punctatissimum      TCTCTACAATAT-TTAATGTAGTAAGAAATCACCAAAA-TTTGATTTCTT
Neoniphon_opercularis            GATTT--AGAATCTTAATGTAATAAGAAATCACCAAAACTTTGATTACTA
Myripristis_kuntee               AGATCAAAAT-C-TGGATGCAGTAAGAAACCACCAACC-GGTGATTTCTG
Myripristis_violacea             AGATCAAAAT-C-TGGATGCAGTAAGAAACCACCAACC-GGTGATTTCTA
Myripristis_murdjan              GAATAAGAATAT-TTAATGTAGTAAGAAACCAGCAATC-GGTGATTCCTG
Myripristis_berndti              AAATAAGAATAT-TTAATGTAGTAAGAAACCAGCAACC-GGTGATTCCTC
Myripristis_vittata              AAGAACAATTAT-TTAATGTAGTAAGAAACCACCAACC-GGTGATTTCTA
Ostichthys_japonicus             GAACACAAATTT-TTAATGTAGTAAGAACCTACCAACC-GGTGATTTCTA
                                              *  *** * *****    * **      ***** **

Sargocentron_caudimaculatum      AATGCATTATA-TTCTTGATGGTCAGGGACAA-TAGAC-TGTGGGGGTA-
Sargocentron_spiniferum          AATGCATTGTA-TTCTTGATGGTCAGGGACAA-TAGAC-TGTGGGGGTA-
Sargocentron_melanospilos        AATGCATACGG-TTCTTGATGGTCAGGGACAA-TA-AT-TGTGGGGGTT-
Sargocentron_rubrum              AATGTACACGG-TTCTTGATGGTCAGGGACAA-TA-AT-TGTGGGGGTT-
Neoniphon_sammara                AATGCATGCGAGTTAATGATGGTCAAGGACAAATAAAA-TGTGGGGGTA-
Sargocentron_diadema             AATGCATACTA-TTATTGATGGTCAGGGACAA-TAACA-TGTGAGGGTT-
Sargocentron_punctatissimum      AATGCATATTA-TTCGTGATAGTCAAGGACA--TCAAT-CGTGAGGGTA-
Neoniphon_opercularis            AATGCATATGCTTTAATGATAATCAAGGGCAAGACACT-AACAAGAATTT
Myripristis_kuntee               AAGGTACTCGG-TTCTTGATAGTCAAGGGCAGGCAAGCATGTGGGGGTA-
Myripristis_violacea             AAGATACTCGG-TTCTTGATGGTCAAGGGCAGGCAAGCATGTGGGGGTA-
Myripristis_murdjan              AATGTATATCA-ATCATGATAATCACGGACAATAAAC--CGTGGGGGTA-
Myripristis_berndti              AATGTATATCA-ATCATGATAATCACGGACAACAAAG--TGTGGGGGTA-
Myripristis_vittata              AATGTATCTAG-TCCTTGATGGTCAGGGACAGTAATC---GTGGGGGTT-
Ostichthys_japonicus             ATTGCATACGG-TTCTTGATGGTCAGGGACAGAAATT---GTGGGGGTT-
                                 *    *          ****  *** ** **             *  *
```

```
Sargocentron caudimaculatum      -GCACTTAACTGAACTATTCCTGGCATTTGGTTCCTACTTCAGGGCCATT
Sargocentron spiniferum          -GCACTTAACTGAATTATTCCTGGCATTTGGTTCCTAATTCAGGGTCATT
Sargocentron melanospilos        -TCACTTGATTGAACTATTCCTGGCATTTGGTTCCTATTTCAGGAACA--
Sargocentron rubrum              -TCACTTGATTGAACTATTCCTGGCATTTGGTTCCTATTTCAGGAACA--
Neoniphon sammara                -GCACTTTGATGAACTATTACTGGCATTTGGTTCCTATTTCAGGTACAT-
Sargocentron diadema             -TCACTTATTTGAATTATTCCTGGCATTTGGTTCCTATTTCAGGGCCATA
Sargocentron punctatissimum      -GCACTTTAATGAATTATTACTGGCATTTGGTTCCTATTTCAGGGCCAT-
Neoniphon opercularis            GGCATTTGAATGAACTATTACTGGCATCTGGTTCCTATTTCAGGTCCAT-
Myripristis kuntee               -ACACTT-AATGATCTATTACTGGCATCTGGTTCCTATTTCAGGTATAA-
Myripristis violacea             -ACACTT-AATGATCTATTACTGGCATCTGGTTCCTATTTCAGGTACAA-
Myripristis murdjan              -ACACTA-AATGATCTATTACTGGCATCTGGTTCCTACTTCAGGCACA--
Myripristis berndti              -ACACTG-AGTGATCTATTACTGGCATCTGGTTCCTATTTCAGGTACAA-
Myripristis vittata              -TCACTT-AGTGAACTATTCCTGGCATTTGGTTCCTACTTCAGGTCCAT-
Ostichthys japonicus             -TCACTT-AGTGAACTATTCCTGGCATTTGGTTCCTATTTCAGGTCCAT-
                                   ** *    ***  **** ******* ********* ******   *

Sargocentron_caudimaculatum      TAAACTGTAAACAATCCCCATA-TTAATGGACTTCAGCAGGCATAAGTTA
Sargocentron_spiniferum          TAGGTTGCAATGAATCCCCATA-TAAATGAACTTCAGCAGGCATAAGTTA
Sargocentron_melanospilos        TAACAAACCTTTAATCCCC-TA-TATCTCACATTTTCCAGGCATAAGTTA
Sargocentron_rubrum              TAATAAACCTTTAATCCCC-TA-TATCCTACATTTTCCAGGCATAAGTTA
Neoniphon_sammara                --AACCATAATCAAACCCCAT--TGCATGAACTTTATCAGGCATAAGTTA
Sargocentron_diadema             TTAATGAAAAT-ATTCCCCATA-ATAATTAATTTT-CCAGGCATAAGTTA
Sargocentron_punctatissimum      ---AATACTGTAAGTACCCCTA-AATATGAATTTTATCAGGCATAAGTTA
Neoniphon_opercularis            AAACACACAAGTATTCCCCATAACAAGTGAATTTTATCAGGCATAGGTTA
Myripristis_kuntee               TTTGA-AGCTTCAATCCCCATA-CAATCTCTTTCTAAGGGGCATAAGTTA
Myripristis_violacea             TTTGA-AGTCCCGATCCCCATA-CAATTTCTTTCTAAAGGGCATAAGTTA
Myripristis_murdjan              -TAAA-AGTAATAGTCCCCATA-AAA-CCCCTTCTAAAGGGCATAAGTTA
Myripristis_berndti              ATGAA-GGTAATAACCCCTACA-GAA-CTCTTTCTAAGGGGCATAAGTTA
Myripristis_vittata              AAAA--AGTAAAAACCCCTAAA-AAG-TTCCTACCAGCGAGCATAAGTTA
Ostichthys_japonicus             TAATTTGGTAACATTCCCCATT-TCA-ATGCTCGTTCCAGGCATAAGTTA
                                                 **                      ***** ****

Sargocentron_caudimaculatum      ATGGTGGTAATACATA--TTAACCTTTAC-CCCACATGCCGGGCATTCTC
Sargocentron_spiniferum          ATGGTGGTAATACATA--TTAACCTTTAC-CCCACATGCCGGGCATTCTC
Sargocentron_melanospilos        ATGGTGGTAATACATA--TTAACCTTTAC-CCCCCATGCCGAGCGTTCTT
Sargocentron_rubrum              ATGGTGGTAATACATA--TTAACCTTTAC-CCCCCATGCCGAGCGTTCTT
Neoniphon_sammara                ATGGTGGTAATACATAAGTTAACCTTTAC-CCCACATGCCGGGCGTTCCT
Sargocentron_diadema             ATGGTGGAATTACATA--TTAACCTTTAC-CCCACATGCCGAGCACTCTT
Sargocentron_punctatissimum      ATGGTGG-AGTACATAA-TAAACCTTTAC-CCCACATGCCGAGCATTCTC
Neoniphon_opercularis            ATGGTGGAATTACATA--TGAACCATTAAACCCACATGCCGGGCATTCTT
Myripristis_kuntee               ATGGTGG-CGTACATAA-ATACCCTTTAC-CCCACATGCCGAGCACTCTT
Myripristis_violacea             ATGGTGG-CGTACATAA-ATACCCTTTAC-CCCACATGCCGAGCACTCTT
Myripristis_murdjan              ATGGTGG-AGTACATAG-TTACCCTTTAC-CCCACATGCCGGGCGCTCTT
Myripristis_berndti              ATGGTGG-AGTACATAG-TCACCCTTTAC-CCCACATGCCGGGCGCTCTT
Myripristis_vittata              ATGGTGG-AGAACATA--TTACCCTTTAC-CCCACATGCCGGGCACTCTT
Ostichthys_japonicus             ATGGTGG-AAAACATA--TTACCCGTTAC-CCAGCATGCCGAGCACTCTT
                                 *******    *****    * ** ***  **  ******* **  **
```

```
Sargocentron_caudimaculatum      GCC-AAAGGGCAGTTGGTTCTCCTTTTTTAGCTTCCTTTCACCTTACATT
Sargocentron_spiniferum          GCT-AAGGGGCAGTTGGTTTTCCTTTTTTAGCTTCCTTTCACCTTACATC
Sargocentron_melanospilos        TCT-AAGGGGCAACGGGTTTTCCTTTTT-AGCTTCCTTTCACCTTACATT
Sargocentron_rubrum              TCT-AAGGGGCAACGGGTTTTCCTTTTT-AGCTTCCTTTCACCTTACATC
Neoniphon_sammara                TCT-AAGGGACAATTGGTTAATCTTTTTTAGCTTCCTTTCATTTTACATC
Sargocentron_diadema             TCCCGAGGGGCAAGGGGTTTTCCTTTTT-AGCTTCCTTTCACTTTACATC
Sargocentron_punctatissimum      TCC-AAAGGGCAATTGGTATTTCTTTTTTAGCTTCCTTTCACTTTACATT
Neoniphon_opercularis            TCC-AAAGGACAATGGGTTTATAATTTTTAGCTTCCTTTCACTTGACATC
Myripristis_kuntee               TCT-AAAGGGTAAGGGGTTTTACCTTTTCCGCTCCCTTTCATTTTACATT
Myripristis_violacea             TCT-AAAGGGTAAGGGGTTTTACTTTTTCCGCTCCCTTTCATTTTACATT
Myripristis_murdjan              TCT-AAAGGGTAAGGGGTTTACCTTTTTTCGCTCCCTTTCATTTCACATC
Myripristis_berndti              TCT-AAAGGGTAAGGGGTTTACCTTTTTTCGCTCCCTTTCATTTTACATC
Myripristis_vittata              TCT-AATGGGCAACGGGTTTACCTTTTTCCGCTCCCTTTCATCTTACATC
Ostichthys_japonicus             TCT-AAAGGGCAAGGGGTTC-TCTTTTTTAGCTTCCTTTCATCTTACATC
                                  *   * **  *   ***      ****  *** *******  * ****

Sargocentron_caudimaculatum      CCGGTGCA--ACGGCAAA-TAACTTAATGAAGGTTGAACATAC-TTTATG
Sargocentron_spiniferum          CCAGTGCA--ACGATAAAATAACTGAATGAAGGTTGAACATATATTTATA
Sargocentron_melanospilos        CCGGTGCG--GCGGCAGA-AGGTAAACTCAAGGTTGAACAT---TTTCT-
Sargocentron_rubrum              CCGGTGCA--GCGGCAGA-AGGTAAACTCAAGGTTGAACAT---TTTCT-
Neoniphon_sammara                CCCGTGCATAACGAAAACATTTCAAAGTGAAGGTAGAACAT---TTCCT-
Sargocentron_diadema             CCGGTGCA--ATGTCAATAATTAATAAT-AAGGTTGAACATAT-TATCTG
Sargocentron_punctatissimum      CCGGTGCG--AAGGATAAAATGATGATTTAAGGTAGTTCATAA--TCCT-
Neoniphon_opercularis            CCAGTGCAAAGCTAAGACAAACGTAAGTTAAGGTTGAACATT--TACCT-
Myripristis_kuntee               CCGGTGCA---CCC-TAAAAATGAAAGCCAAGGTAGAACATA--TTCCT-
Myripristis_violacea             CCGGTGCA---CCC-TAAAAATGAAAGTCAAGGTAGAACATA--TTCCT-
Myripristis_murdjan              CCGGTGCA---AAG-AAAAATTTAAT-TGGAGGTAGAACATG--TTCCT-
Myripristis_berndti              CCAGTGCA---AAG-GAAGGACTGAA-TGGAAGTAGAACATG--TTCCT-
Myripristis_vittata              CCAGTGCA---GCGCTAAGGATAAAAATCAAGGTAGAGCATT--TCCTT-
Ostichthys_japonicus             CCAGTGCA---GCGCTAAGGTTAACTAATAAGGTAGAACATT--TTCTT-
                                 ** ****                       * ** *  ***       *

Sargocentron_caudimaculatum      TTGATATAATAACAAGTAATGTATTCTCCGGGCAGGAATATG----CATG
Sargocentron_spiniferum          ATGGTGTAATAACAAATAATGTTTTCCCCGGGCAA-AATGTG----CATG
Sargocentron_melanospilos        ---------------------TGCTTGCAAGGAAATAGTATT----CATG
Sargocentron_rubrum              ---------------------TGCTTGCAAGGAAATAGTATT----CATG
Neoniphon_sammara                -------------------TGTATTCCGGAGGTAATGGTGGTA-T-CATG
Sargocentron_diadema             --------------------GTGTAAATAAAGAAATA-TGTAT-T-CATG
Sargocentron_punctatissimum      -----------------------TGCCAGAGTTAATGATAGTA-TTCATG
Neoniphon_opercularis            -------------------TGCATTCGTAAAACGTAAACAATAATTCATG
Myripristis_kuntee               ---------------------TGTAACATAAGATACACTTTT----AATG
Myripristis_violacea             ---------------------TGTAACACAAGACACACTTTT----AATG
Myripristis_murdjan              ---------------------TGCT-CGCGTAAAATATGTTT----AGTG
Myripristis_berndti              ---------------------TGCC-CGCATAAA-TACGTTT----AATG
Myripristis_vittata              ---------------------GCACGCACAGGAAATATTATT----CGTG
Ostichthys_japonicus             ---------------------GCTCGCA-AGCGAATAGTATT----CATG
                                                                                **
```

```
Sargocentron_caudimaculatum      -GTGTTAAAACTTATA----TAATCAA-TGAAACCACAAATAGGGATATC
Sargocentron_spiniferum          -GTGTTAAAACTTATA----TCATTAA-TGAATCCACAAATAAGGATATC
Sargocentron_melanospilos        -GTGGTTAGATTTA------TTATTAA-AGAA-CCACATTATTAGATATC
Sargocentron_rubrum              -GTGGTTAGATTTA------TCGTTAA-AGAA-CCACATTATTAGATATC
Neoniphon_sammara                TATGAGAAGGTTTGTAA---CAATTAA-TGATCCCACATATTTAGATATC
Sargocentron_diadema             -GTGGAAAGATTTG------TTTTTAA-GAAT--TACATATTAAGATATC
Sargocentron_punctatissimum      -ATGATAAACTATT-------CACCAA-ATAA-CCACATATTAGGATATC
Neoniphon_opercularis            TTAATAAAGGCTTAAAATAATCATTAA-CAAAACCACATATTAAGATATC
Myripristis_kuntee               -GAGTAAAGGATGGAA-----AGTAAT---ATCTCACATAAAGTAATATC
Myripristis_violacea             -GAGTAAAGAATGGAA-----AGTAAT---ATCTTGCATAAAGTGATATC
Myripristis_murdjan              -GAGGGAAGACTAGAA-----GGAAAGTAGAGTTTGCATGTAATGGATTC
Myripristis_berndti              -GAGGTAGGGCTGAAA-----GAAAAGCAAGACTTGCATGTAATGGATTC
Myripristis_vittata              -GAGGTAAGATTTATA-----AATGAA--AAATCCACATACTAATATTTC
Ostichthys_japonicus             -GTGGAAAGATTTT-------AATTAA--ATAACCACATATTAGGATATC
                                                          *          **          **

Sargocentron_caudimaculatum      AAGTGCATAAGTAATTTTT-TACCTCGAGCAATTTCACCCAATAATATTG
Sargocentron_spiniferum          AAGTGCATAAATAATTTTT-TACCTCGAGCAATTTCACCCAATAATATTG
Sargocentron_melanospilos        AAGTGCATAATAATTTTTT-TTTTCTAGGCAAAT--ACCTAATA-TATCG
Sargocentron_rubrum              AAGTGCATAATAATTTTTT-TTTTCTAGGCAAAT--ACCTAATA-TATCG
Neoniphon_sammara                AAGTGCATAAT--ATTTTT-TTTTCTAGACAAT---ATTCTATGATATTT
Sargocentron_diadema             AAGGACATAAATAACTTTTATTAACTAGGCATAT--ATCTAATA-TATGA
Sargocentron_punctatissimum      AAGTGCATAACATATTTCT--TTACTAGGCAAAT--ATCCAACA-TATCA
Neoniphon_opercularis            AAGTGCATAACAATTTTTTTATTTCTAGGCAAAT--ATCCTATA-TATCA
Myripristis_kuntee               AAGTGCATAATATATTTATTCTCAATAGGCAAAG--CTCTAACA-CACCA
Myripristis_violacea             AAGTGCATAATATATTTATTTTCAATAGGCAAAG--CTCTAACA-CACCA
Myripristis_murdjan              AAGTGCATAATATATTT-TTATTACTAGGCAAAA--CTCTAACA-CACCA
Myripristis_berndti              AAGTGCATAATATATTT-TTACTACCAGGCAAAA--CTCTAACA-CACCA
Myripristis_vittata              ATGTGCATAATATACTTTTATTTCTCGGACAAAT--CCCTA-TA-TATCA
Ostichthys_japonicus             ATGTGCATAATAT-TTTTTATTTTCTAGGCAAAT--ACCTAATA-TATTA
                                 * *  *****     ** *          **               *

Sargocentron_caudimaculatum      CCCCCTTCGTTTTTGCGCGTTAAACCCCCC-TACCCCCC-AACACCCCTA
Sargocentron_spiniferum          CCCCCTCCGTTTTTGCGCGTTAAACCCCCC-TACCCCCC-AACACCCCTA
Sargocentron_melanospilos        CCCC-TCCGTTTTTGCGCGTTAAACCCCCC-TACCCCCCTAACACTCCTG
Sargocentron_rubrum              CCCC-TCCGTTTTTGCGCGTTAAACCCCCC-TACCCCCCTAACACTCCTA
Neoniphon_sammara                CCCC-CTGGGTTTTGCGCGTTAAACCCCCC-TACCCCCC-AATACTCCTA
Sargocentron_diadema             CCCCCTCGGTTTTTGCGCGT-AAACCCCCC-TACCCCCC-AATACTCCTG
Sargocentron_punctatissimum      CCCC-CTGGTTTTTGCGCGT-AAACCCCCC-TACCCCCC-AACACTCCTG
Neoniphon_opercularis            CCCC-CTGGTTTTTGCGCGTTAAACCCCCC-TACCCCCC-TATACTCCTG
Myripristis_kuntee               CCCC-CTGGCTTTTTCGCGTTAAACCCCCC-TACCCCCC-TACACCCCTG
Myripristis_violacea             CCCC-CTGGCTTTTTCGCGTTAAACCCCCC-TACCCCCC-TACACCCCTG
Myripristis_murdjan              CCCC-CTGGTTTTTTCGCGTTAAACCCCCCCTACCCCCC-TACACTCCTG
Myripristis_berndti              CCCC-CTGGTTTTTTCGCGTTAAACCCCCCCTACCCCCC-TACACTCCTG
Myripristis_vittata              CCCC-CTGGTTTTTTCGCGT-AAACCCCCC-TACCCCCC-TACACCCCTG
Ostichthys_japonicus             CCCC-TCGGCTTTTGCGCGTCAAACCCCCC-TACCCCCC-TACACTCCTG
                                 ****    * **** ***** ********* ********  * ** ***
```

```
Sargocentron_caudimaculatum      AGATCTCTATCACTCCTGTAAACCCCCCGGAAACAGGATAAACCTTCGGG
Sargocentron_spiniferum          AGATCTCTATCACTCCTGTAAACCCCCCGGAAACAGGATAAACCTTCGGG
Sargocentron_melanospilos        AGATCTATATTATTCCTGTAAACCCCCCGGAAACAGGA--AAACCTCGAG
Sargocentron_rubrum              AGATCTATATTATTCCTGTAAACCCCCCGGAAACAGGA--AAACCTCGAG
Neoniphon_sammara                AGATTCCTAATATTCCTGTAAACCCCC-GAAAACAGGACTAAACCTCGAG
Sargocentron_diadema             AGATTACTATTATTCCTGTAAACCCCCCGGAAACAGGATCTAACCCCGAG
Sargocentron_punctatissimum      AGATCGCTATTATTCCTGCAAACCCCCCGGAAACAGGAC--AACCTCGAG
Neoniphon_opercularis            AGATCACTGTCATTCCTGCAAACCCCC-GAAAACAGGACTAAACCTCGAG
Myripristis_kuntee               AGACCTCTATCACTCCTGCAAACCCCCCGGAAACAGGAC-AAATCTCAAA
Myripristis_violacea             AGACCTCTATCACTCCTGCAAACCCCCCGGAAACAGGAC-AAATCTCAAA
Myripristis_murdjan              AGATCCTTATCACTCCTGCAAACCCCCCGGAAACAGGAC-AAACCTCAAA
Myripristis_berndti              AGATCCTTATCACTCCTGCAAACCCCCCGGAAACAGGAC-AAACCTCAAA
Myripristis_vittata              AGACCTCTATCACTCCTGCAAACCCCCCGGAAACAGGAA-AAGTCTCAAA
Ostichthys_japonicus             AGATCTTTATTACTCCTGCAAACCCCCCGGAAACAGGAA-A-GTCTCAAG
                                 ***    *   * ***** ******** * ********        *

Sargocentron_caudimaculatum      CATTG--------CTACTTCCCCATCAAATTTGCGTCTATCACAAT----
Sargocentron_spiniferum          CATTG--------CTACTTCACCATCAAATTTGCGTCTATCACAAT----
Sargocentron_melanospilos        TGTTA--------ATTATCCTCCCT--AATTTATGTCTATTACAAT----
Sargocentron_rubrum              TGTTA--------ATTATCCTCCCT--AATTTATGTCTATTACAAT----
Neoniphon_sammara                TATTA--------ACCTATTTTAATTCAAATTATATTTACTTAAATTGTA
Sargocentron_diadema             TATTA--------AATATTCCCAGCATAAATTATGCTTATTTATATT---
Sargocentron_punctatissimum      TGTTATA------AGCCTCCCTAATCTAAATTACACTCATTTACATT---
Neoniphon_opercularis            TACTAAT------TCACTCCCAATTCAAAAATAT-TTTACTTATATT---
Myripristis_kuntee               CAGTGTTCTTAATATTCCAACCACATTTATTTGTGCTTATTTACATT---
Myripristis_violacea             CAGTGTTCTTTATACTCCAACCACATTTATTTGTGCTTATTTACATT---
Myripristis_murdjan              CAGTGCTATTAATCTTCTAACCACATTTATTTGTGCTTATTTACATT---
Myripristis_berndti              CAGTGCTATTAATCTTCTAACCACATTTATTTGTGCTTATTTACATT---
Myripristis_vittata              TAGTGCTATT--TTTTTTAACCACATCTATTTATGCTTATTTACATT---
Ostichthys_japonicus             TGATA----------TTTAAGTAATTCAATTTATGTATATTTACATT---
                                    *                        *  *      *     **

Sargocentron_caudimaculatum      ---ATTTCAATATTG-CACATTTTG-------------------------
Sargocentron_spiniferum          ---ATTTCAATATTG-CACATTTT--------------------------
Sargocentron_melanospilos        ---ATTTCAATATTA-CACATTTTG-------------------------
Sargocentron_rubrum              ---ATTTCAATATTA-CACATTTT--------------------------
Neoniphon_sammara                ATAATTACAATATTAACCTATTTTAATTCAAATTATATTTWCTTWAATTG
Sargocentron_diadema             ---ATTATAATATTG-CACATAG---------------------------
Sargocentron_punctatissimum      ---ATTATAATATTG-CACATGG---------------------------
Neoniphon_opercularis            ---ATTACAGTATTGCAGCATTACAGTACTACAACATTTTTATAATG--
Myripristis_kuntee               ---ATTATAATATTA-CACACAG---------------------------
Myripristis_violacea             ---ATTATAATATTA-CACACAG---------------------------
Myripristis_murdjan              ---ATTATAATATTA-CACACAG---------------------------
Myripristis_berndti              ---ATTATAATATTA-CACACA----------------------------
Myripristis_vittata              ---ATTATAATATTA-CACACA----------------------------
Ostichthys_japonicus             ---ATTATAATATTG-CACACA----------------------------
                                    ***  * ****     *
```

```
Sargocentron_caudimaculatum      AGATCTCTATCACTCCTGTAAACCCCCCGGAAACAGGATAAACCTTCGGG
Sargocentron_spiniferum          AGATCTCTATCACTCCTGTAAACCCCCCGGAAACAGGATAAACCTTCGGG
Sargocentron_melanospilos        AGATCTATATTATTCCTGTAAACCCCCCGGAAACAGGA--AAACCTCGAG
Sargocentron_rubrum              AGATCTATATTATTCCTGTAAACCCCCCGGAAACAGGA--AAACCTCGAG
Neoniphon_sammara                AGATTCCTAATATTCCTGTAAACCCCC-GAAAACAGGACTAAACCTCGAG
Sargocentron_diadema             AGATTACTATTATTCCTGTAAACCCCCCGGAAACAGGATCTAACCCCGAG
Sargocentron_punctatissimum      AGATCGCTATTATTCCTGCAAACCCCCCGGAAACAGGAC--AACCTCGAG
Neoniphon_opercularis            AGATCACTGTCATTCCTGCAAACCCCC-GAAAACAGGACTAAACCTCGAG
Myripristis_kuntee               AGACCTCTATCACTCCTGCAAACCCCCCGGAAACAGGAC-AAATCTCAAA
Myripristis_violacea             AGACCTCTATCACTCCTGCAAACCCCCCGGAAACAGGAC-AAATCTCAAA
Myripristis_murdjan              AGATCCTTATCACTCCTGCAAACCCCCCGGAAACAGGAC-AAACCTCAAA
Myripristis_berndti              AGATCCTTATCACTCCTGCAAACCCCCCGGAAACAGGAC-AAACCTCAAA
Myripristis_vittata              AGACCTCTATCACTCCTGCAAACCCCCCGGAAACAGGAA-AAGTCTCAAA
Ostichthys_japonicus             AGATCTTTATTACTCCTGCAAACCCCCCGGAAACAGGAA-A-GTCTCAAG
                                 ***    *   * ***** ******** * ********        *

Sargocentron_caudimaculatum      CATTG--------CTACTTCCCCATCAAATTTGCGTCTATCACAAT----
Sargocentron_spiniferum          CATTG--------CTACTTCACCATCAAATTTGCGTCTATCACAAT----
Sargocentron_melanospilos        TGTTA--------ATTATCCTCCCT--AATTTATGTCTATTACAAT----
Sargocentron_rubrum              TGTTA--------ATTATCCTCCCT--AATTTATGTCTATTACAAT----
Neoniphon_sammara                TATTA--------ACCTATTTTAATTCAAATTATATTTACTTAAATTGTA
Sargocentron_diadema             TATTA--------AATATTCCCAGCATAAATTATGCTTATTTATATT---
Sargocentron_punctatissimum      TGTTATA------AGCCTCCCTAATCTAAATTACACTCATTTACATT---
Neoniphon_opercularis            TACTAAT------TCACTCCCAATTCAAAAATAT-TTTACTTATATT---
Myripristis_kuntee               CAGTGTTCTTAATATTCCAACCACATTTATTTGTGCTTATTTACATT---
Myripristis_violacea             CAGTGTTCTTTATACTCCAACCACATTTATTTGTGCTTATTTACATT---
Myripristis_murdjan              CAGTGCTATTAATCTTCTAACCACATTTATTTGTGCTTATTTACATT---
Myripristis_berndti              CAGTGCTATTAATCTTCTAACCACATTTATTTGTGCTTATTTACATT---
Myripristis_vittata              TAGTGCTATT--TTTTTTAACCACATCTATTTATGCTTATTTACATT---
Ostichthys_japonicus             TGATA----------TTTAAGTAATTCAATTTATGTATATTTACATT---
                                    *                        *  *      *     **

Sargocentron_caudimaculatum      ---ATTTCAATATTG-CACATTTTG-------------------------
Sargocentron_spiniferum          ---ATTTCAATATTG-CACATTTT--------------------------
Sargocentron_melanospilos        ---ATTTCAATATTA-CACATTTTG-------------------------
Sargocentron_rubrum              ---ATTTCAATATTA-CACATTTT--------------------------
Neoniphon_sammara                ATAATTACAATATTAACCTATTTTAATTCAAATTATATTTWCTTWAATTG
Sargocentron_diadema             ---ATTATAATATTG-CACATAG---------------------------
Sargocentron_punctatissimum      ---ATTATAATATTG-CACATGG---------------------------
Neoniphon_opercularis            ---ATTACAGTATTGCAGCATTACAGTACTACAACATTTTTTATAATG--
Myripristis_kuntee               ---ATTATAATATTA-CACACAG---------------------------
Myripristis_violacea             ---ATTATAATATTA-CACACAG---------------------------
Myripristis_murdjan              ---ATTATAATATTA-CACACAG---------------------------
Myripristis_berndti              ---ATTATAATATTA-CACACA----------------------------
Myripristis_vittata              ---ATTATAATATTA-CACACA----------------------------
Ostichthys_japonicus             ---ATTATAATATTG-CACACA----------------------------
                                    ***  * ****     *
```

Sargocentron_caudimaculatum	--------
Sargocentron_spiniferum	--------
Sargocentron_melanospilos	--------
Sargocentron_rubrum	--------
Neoniphon_sammara	TTCACATA
Sargocentron_diadema	--------
Sargocentron_punctatissimum	--------
Neoniphon_opercularis	--------
Myripristis_kuntee	--------
Myripristis_violacea	--------
Myripristis_murdjan	--------
Myripristis_berndti	--------
Myripristis_vittata	--------
Ostichthys_japonicus	--------
